# Neutron crystallographic analysis of the nucleotide-binding domain of Hsp72 in complex with ADP

**DOI:** 10.1107/S2052252522006297

**Published:** 2022-07-16

**Authors:** Takeshi Yokoyama, Shiho Fujii, Andreas Ostermann, Tobias E. Schrader, Yuko Nabeshima, Mineyuki Mizuguchi

**Affiliations:** a Faculty of Pharmaceutical Sciences, University of Toyama, 2630 Sugkitani, Toyama 930-0914, Japan; b Heinz Maier-Leibnitz Zentrum (MLZ), Technische Universtät München, Lichtenbergstrasse 1, 85748 Garching, Germany; c Forschungszentrum Jülich GmbH, Jülich Centre for Neutron Science (JCNS) at Heinz Maier-Leibnitz Zentrum (MLZ), Lichtenbergstrasse 1, 85748 Garching, Germany; University of Michigan, USA

**Keywords:** neutron protein crystallography, heat-shock proteins, Hsp72, hydrogen-bond networks, molecular chaperones, ATPases, water clusters

## Abstract

The 70 kDa heat-shock proteins (Hsp70s) are ATP-dependent molecular chaperones that contain an N-terminal nucleotide-binding domain (NBD) and a C-terminal substrate-binding domain. Hsp70s bind to misfolded/unfolded proteins and thereby prevent their aggregation. The ATP hydrolysis reaction in the NBD plays a key role in allosteric control of the binding of substrate proteins. In the present study, the neutron crystal structure of the NBD of Hsp72, a heat-inducible Hsp70 family member, was solved in complex with ADP in order to study the structure–function relationship with a focus on hydrogens. ADP bound to Hsp72 was fully deprotonated, and the catalytically important residues, including Asp10, Asp199 and Asp206, are also deprotonated. Neutron analysis also enabled the characterization of the water clusters in the NBD. Enzymatic assays and X-ray crystallographic analysis revealed that the Y149A mutation exhibited a higher ATPase activity and caused disruption of the water cluster and incorporation of an additional magnesium ion. Tyr149 was suggested to contribute to the low intrinsic ATPase activity and to stabilize the water cluster. Collectively, these structural studies will help to elucidate the molecular basis of the function of Hsp72.

## Abstract 2

The neutron crystallographic structure of an Hsp72–ADP complex is reported.

## 1. Introduction

The 70 kDa heat-shock proteins (Hsp70s) are widely conserved molecular chaperones in eukaryotes. Hsp70s are expressed in response to a variety of cellular stresses such as heat, UV irradiation and oxygen deprivation, and play key roles in regulation of cellular protein homeostasis by protein refolding, protein translocation across organelles and dis­aggregation of protein aggregates (Hartl *et al.*, 2011 ▸; Mayer & Bukau, 2005 ▸; Saibil, 2013 ▸). In humans, several paralogs of Hsp70 are found in the cytosol, endoplasmic reticulum (ER) and mitochondria. The heat-inducible Hsp70 (Hsp72, also known as HSPA1A and HSPA1B) and the heat-shock cognate Hsp70 (Hsc70, also known as HSPA8) are the major cytosolic Hsp70s, while BiP (HSPA5) and mortalin (HSPA9) are localized in the ER and mitochondria, respectively (Vos *et al.*, 2008 ▸). The *Escherichia coli* Hsp70 ortholog DnaK is one of the most well studied members of the Hsp70 family.

**Figure 1:**
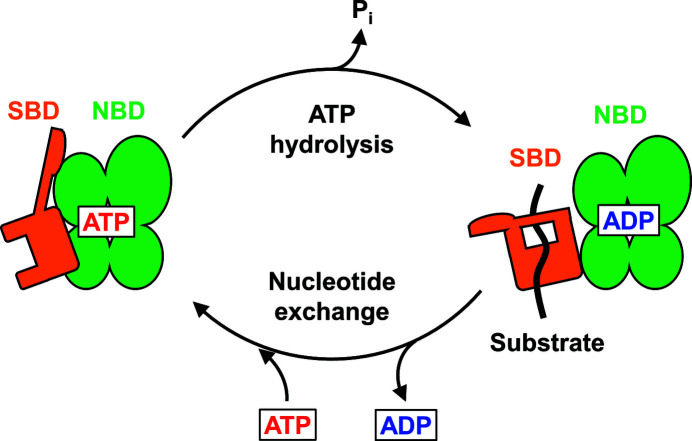
Schematic view of the allosteric regulation of the substrate binding of Hsp70.

Hsp70 is an ATPase that binds to and releases unfolded and misfolded proteins depending on the nucleotide-binding state. Hsp70 is composed of an N-terminal nucleotide-binding domain (NBD), a C-terminal substrate-binding domain (SBD) and a flexible linker that connects the NBD and SBD. The NBD can communicate allosterically with the SBD (Fig. 1 ▸). The binding of ATP to the NBD decreases the substrate-binding affinity of the SBD, and its hydrolysis to ADP increases the substrate binding affinity (Pierpaoli *et al.*, 1998 ▸). An NMR-based structural study of DnaK in complex with ADP and peptide and a crystallographic analysis of DnaK in complex with ATP showed that the ADP-bound state adopts an extended conformation and the binding of ATP in the NBD induces large structural rearrangements (Qi *et al.*, 2013 ▸; Bertelsen *et al.*, 2009 ▸). These structural rearrangements consist of the docking of the SBD and the intradomain linker to the NBD and conformational change in the SBD, resulting in the promotion of substrate protein release (Zuiderweg *et al.*, 2013 ▸). The substrate binding and release cycle can also be controlled by co-chaperones such as Hsp40 and nucleotide-exchange factors (NEFs). The J-domain of Hsp40 stimulates the ATPase activity. NMR and molecular-dynamics simulation studies indicated that the J-domain predominantly interacts with the NBD in the bacterial Hsp70/Hsp40 system (Ahmad *et al.*, 2011 ▸), while it still remains to be seen how Hsp40 binds to Hsp70 (Li *et al.*, 2009 ▸; Alderson *et al.*, 2016 ▸). The eukaryotic NEF Bcl2-associated anthanogene 1 (BAG1) protein binds directly to the NBD of Hsp70 and stimulates the ATPase activity. The binding of BAG1 to Hsc70 induces opening of the nucleotide-binding cleft, promoting the dissociation of nucleotides (Sondermann *et al.*, 2001 ▸).

**Figure 2:**
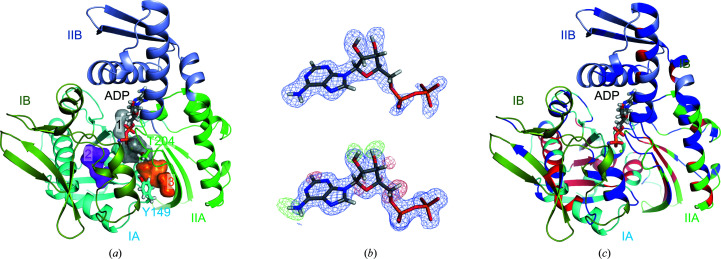
(*a*) Ribbon plot of the overall joint XN structure of the NBD of Hsp72 in complex with ADP (PDB entry 7f4x). Subdomains IA, IB, IIA and IIB are coloured cyan, split pea, green and light blue, respectively. The ADP molecule and the side chains of Thr204 and Tyr149 are shown as stick models. Water clusters 1, 2 and 3 are shown as stick and semi-transparent surface models in grey, magenta and orange, respectively. (*b*) Top: the |*F*_o_| − |*F*_c_| neutron scattering length density map omitting ADP contoured at +4σ. Bottom: the |*F*_o_| − |*F*_c_| electron-density map omitting ADP contoured at +4σ (blue) and the |*F*_o_| − |*F*_c_| neutron scattering length density map omitting H and D atoms contoured at +3.3σ (green) and −3.3σ (red). (*c*) The H/D-exchanged and unexchanged regions are projected in the ribbon model. The H/D-exchanged and unexchanged regions are coloured blue and red, respectively.

Hydrolysis of ATP to ADP in the NBD triggers functionally essential cycles of substrate binding and release, and thus the NBD plays a pivotal role during the cycle. X-ray crystal structures of the isolated NBDs of human Hsp70 paralogs, including Hsp72, Hsc70, BiP and mortalin, have been determined (Shida *et al.*, 2010 ▸; Zhang *et al.*, 2015 ▸; Wisniewska *et al.*, 2010 ▸; Amick *et al.*, 2014 ▸). Their overall structures are well conserved, with sequence identities of 52–89%. The NBD is composed of four subdomains, IA, IB, IIA and IIB, and the nucleotide-binding pocket is located in the central part of the subdomains (Fig. 2 ▸ *a*). Crystallographic analysis showed that ADP binds to Hsp70 simultaneously with catalytic magnesium ions, orthophosphate ions and monovalent cations such as sodium and potassium ions depending on the purification buffer or crystallization additives, while the ATP analog AMPPnP only binds to Hsp70 with a catalytic magnesium ion (Shida *et al.*, 2010 ▸; Arakawa *et al.*, 2011 ▸). The monovalent ions are required for optimal ATPase activity (Wilbanks & McKay, 1995 ▸; O’Brien & McKay, 1995 ▸), and the presence of orthophosphate ions increases the binding affinity of Hsp70 for ADP (Arakawa *et al.*, 2011 ▸). The ATPase reaction is a hydrolysis reaction using a water molecule as a nucleophile. The enzymatic catalysis can therefore be affected not only by the binding affinity for ATP but also by various conditions such as the static stability, water accessibility and long-range electrostatic interactions. To date, X-ray crystallographic, biophysical and mutagenic analyses of the NBD of Hsp70 have been widely conducted to investigate the molecular features that are involved in the intrinsic ATPase activity (Arakawa *et al.*, 2011 ▸; O’Brien *et al.*, 1996 ▸; O’Brien & McKay, 1993 ▸; Ha *et al.*, 1997 ▸; Moseng *et al.*, 2019 ▸). In addition to these, we considered that neutron protein crystallography would be a good approach to understand the molecular details of this ATPase from a different perspective.

Neutron protein crystallography is a useful technique to determine the positions of H and D atoms (O’Dell *et al.*, 2016 ▸). Neutrons are scattered from nuclei. While the neutron scattering length for deuterium is comparable to those for carbon, nitrogen and oxygen, H atoms exhibit a negative scattering length with half of the magnitude of the scattering length of D atoms. Therefore, deuterium can be directly observed at moderate resolution, but H atoms are difficult to visualize at resolutions lower than 2.2 Å. Hydrogen/deuterium-exchanged or perdeuterated crystals are generally used for neutron protein crystallography, because H atoms scatter with a strong incoherent scattering component, resulting in high backgrounds (Howard *et al.*, 2016 ▸; Dajnowicz *et al.*, 2017 ▸; Schiebel *et al.*, 2018 ▸; Yokoyama *et al.*, 2019 ▸). Neutron crystallography can therefore determine protonation states by detection of the occupation of acidic sites with deuterium, and it can furthermore find the directions of heavy waters (D_2_O) and the hydrogen/deuterium-exchange ratio even at moderate resolution, although neutron diffraction experiments require larger crystals compared with X-ray diffraction experiments.

In the present study, we describe the neutron crystal structure of the NBD of human major inducible Hsp72 in complex with ADP at 2.2 Å resolution. We fully characterize the protonation states in the ADP-binding pocket, the protein flexibility from the H/D-exchange ratios and two water clusters in the NBD. We also characterize the structures of the hydrogen-bond networks and the water cluster found in the Hsp72–ADP complex. The neutron crystallographic analysis motivated us to investigate the role of Tyr149, which is associated with water cluster 3 and the surrounding hydrogen-bond network. Together with the results of the neutron crystallographic analysis, we discuss the ATPase activity of Hsp72 in relation to the hydrogen-bond networks and water clusters in the protein.

## 2. Materials and methods

### 2.1. Protein production

The expression plasmid for the NBD (residues 1–380) of Y149A-mutated Hsp72 (Y149A-Hsp72) was prepared with the QuikChange Site-Directed Mutagenesis Kit using the pET-22b(+) plasmid for the NBD of wild-type HspA1B (WT-HspA1B) as a template. The expression and purification of WT-Hsp72 and Y149A-Hsp72 were carried out as reported previously (Yokoyama *et al.*, 2017 ▸). In brief, the recombinant plasmid was transformed into *Escherichia coli* BL21(DE3) cells. Protein expression was induced with 0.25 m*M* isopropyl β-d-1-thiogalactopyranoside (IPTG). The cells were incubated at 293 K overnight with agitation at 180 rev min^−1^. The harvested cells were resuspended, lysed by sonication and the cell lysate was centrifuged at 9400*g* for 60 min at 277 K. Metal-affinity chromatography was performed using Ni–NTA and the desired protein fractions were pooled. The protein sample was dialysed against a buffer consisting of 20 m*M* Tris–HCl pH 8.0, 100 m*M* NaCl. The protein sample was then subjected to affinity chromatography using Blue Sepharose 6 Fast Flow resin. The protein was eluted with a buffer consisting of 20 m*M* Tris–HCl pH 8.0, 1 *M* NaCl and the elution fraction was dialysed against a buffer consisting of 20 m*M* Tris–HCl pH 8.0, 100 m*M* NaCl.

The recombinant pET-22b(+) plasmids for full-length Hsp72 (residues 1–641), DnaJ homolog subfamily B member 1 (DnaJB1; residues 1–340) and the BAG domain of BAG family molecular chaperone regulator 1 (BAG1; residues 151–261) were synthesized by GenScript. The plasmids were transformed into *E. coli* SoluBL21 cells and the transformed cells were grown at 310 K to an OD_600_ of 0.4–0.6; expression of the C-terminally 6×His-tagged proteins was then induced overnight at 293 K with 0.25 m*M* IPTG. The cell pellets were lysed by sonication and the supernatant was purified by nickel-affinity chromatography followed by size-exclusion chromatography using a Superdex 200 gel-filtration column equilibrated with a buffer consisting of 20 m*M* Tris–HCl pH 8.0, 100 m*M* NaCl. Protein aliquots were concentrated to 10–20 mg ml^−1^ using an Amicon Ultra-10 centrifugal device (10 kDa cutoff), flash-frozen using liquid nitrogen and stored at 243 K. The expression plasmids for full-length Y149A-mutated Hsp72 and full-length T204A-mutated Hsp72 were prepared with the QuikChange Site-Directed Mutagenesis Kit using the plasmid for full-length WT-Hsp72 as a template. Identical expression and purification schemes were performed for full-length Y149A-mutated Hsp72 and full-length T204A-mutated Hsp72.

### 2.2. ATPase activity measurements

The ATPase enzymatic activities of full-length Hsp72 were assessed by a colorimetric malachite green assay in which orthophosphate ions can directly be detected, as described previously (Yokoyama *et al.*, 2021 ▸). The ATPase reaction was directly performed in microplate wells using a 40 µl sample composed of 0.3 µ*M* full-length Hsp72 (WT, Y149A or T204A), 40 m*M* Tris–HCl pH 8, 50 m*M* KCl, 10 m*M* MgCl_2_ and 15–1000 µ*M* ATP in the presence or absence of the co-chaperones DnaJB1 and BAG1. The reaction samples were incubated at 310 K for 180 min (WT and T204A) or 40 min (Y149A) and were then stained by the addition of 160 µl malachite green reagent composed of one volume of 4.2%(*w*/*v*) ammonium molybdate in 4 *N* HCl and three volumes of 0.045%(*w*/*v*) malachite green. The absorbance at 620 nm was measured using a FilterMax F5 (Molecular Devices). The values of the Michaelis constant *K*_m_ and *k*_cat_ were determined by a Lineweaver–Burk double-reciprocal plot for ATP as a substrate.

### 2.3. X-ray protein crystallography

Crystallization was performed by the hanging-drop vapour-diffusion method at 293 K. Single crystals of the NBD of Y149A-Hsp72 in complex with ADP were obtained by equilibrating a 2 µl droplet composed of 12.6 mg ml^−1^ Y149A-Hsp72 NBD, 2 m*M* ADP, 15.5% polyethylene glycol monomethyl ether 550 (PEG 550 MME), 100 m*M* MgCl_2_, 150 m*M* KCl, 50 m*M* bis-Tris pH 6.0 against 1000 µl crystallization buffer composed of 31% PEG 550 MME, 200 m*M* MgCl_2_, 300 m*M* KCl, 100 m*M* bis-Tris pH 6.0. Although Y149A-Hsp72 in complex with AMPPnP also crystallized under the crystallization conditions used for the Y149A-Hsp72–ADP complex, single crystals appropriate for X-ray diffraction experiments were not obtained. We therefore adopted a micro-seeding method to produce single crystals. The parent crystals were obtained by equilibrating a 2 µl droplet composed of 12.9 mg ml^−1^ Y149A-Hsp72 NBD, 2 m*M* AMPPnP, 14.5% PEG 550 MME, 100 m*M* MgCl_2_, 50 m*M* Tris–HCl pH 7.0 against 1000 µl crystallization buffer composed of 29% PEG 550 MME, 200 m*M* MgCl_2_, 100 m*M* Tris–HCl pH 7.0. The crystals were directly crushed in the droplet using a needle and the seed stock was prepared by 1000-fold dilution of the droplet with additional reservoir solution. Finally, single crystals of Y149A-Hsp72 in complex with AMPPnP were obtained with crystallization conditions consisting of 29% PEG 550 MME, 200 m*M* MgCl_2_, 100 m*M* Tris–HCl pH 7.0 using a droplet composed of 1.9 µl 16.1 mg ml^−1^ Y149A-Hsp72, 1.9 µl reservoir solution and 0.2 µl seed stock. Crystals began to appear within a few hours and stopped growing after one week. The crystals were cryoprotected with 33% PEG 550 MME and directly stored in liquid nitrogen until the X-ray diffraction experiment.

Cryogenic X-ray diffraction data were collected on beamline BL-17A at the Photon Factory (PF) in Japan and were processed with *XDS* (Kabsch, 2010 ▸). The Y149A-Hsp72 crystals belonged to space group *P*2_1_2_1_2_1_ and were isomorphous with the room-temperature WT-Hsp72 crystals. Therefore, structure refinement could be carried out using the coordinates of the room-temperature WT-Hsp72 structure without performing Patterson-based molecular replacement. Several rounds of iterative model building and refinement were performed using *Coot* and *phenix.refine* (Emsley *et al.*, 2010 ▸; Adams *et al.*, 2010 ▸). The coordinates and structure factors of Y149A-Hsp72–ADP and Y149A-Hsp72–AMPPnP have been deposited in the Protein Data Bank as PDB entries 7f4z and 7f50, respectively. The crystal and refinement data are summarized in Table 1 ▸.

### 2.4. Joint X-ray/neutron (XN) refinement

We have previously collected room-temperature neutron and X-ray diffraction data for the nucleotide-binding domain of HspA1B (Hsp72) in complex with ADP at 2.2 and 1.6 Å resolution, respectively (Yokoyama *et al.*, 2017 ▸). Prior to the joint XN refinement, the structure was refined using the X-ray data. The structure was solved by molecular replacement with *MOLREP* (Vagin & Teplyakov, 2010 ▸) using the structure of Hsp72 complexed with an inhibitor (PDB entry 5aqz; Cheese­man *et al.*, 2016 ▸) as a search model. After several cycles of X-ray structure refinement with manual correction in *Coot* (Emsley *et al.*, 2010 ▸), joint XN refinement was initiated from the refined structure using *phenix.refine* (Adams *et al.*, 2010 ▸). The H and D atoms were included using *phenix.ready_set* and heavy water molecules were added manually (Adams *et al.*, 2010 ▸). Omit maps were calculated using *phenix.map* after omitting target atoms from the model (Adams *et al.*, 2010 ▸). The final *R*_cryst_ and *R*_free_ factors for the neutron data were 18.4% and 22.2%, respectively. The final *R*_cryst_ and *R*_free_ factors for the X-ray data were 16.7% and 19%, respectively. The coordinates and structure factors have been deposited in the Protein Data Bank as PDB entry 7f4x. The crystal and refinement data are summarized in Table 2 ▸.

## 3. Results

### 3.1. Overall joint XN structure of WT-Hsp72 in complex with ADP

In the previous study, neutron diffraction data and X-ray diffraction data of the NBD of Hsp72 (residues 1–380) in complex with ADP were collected at room temperature using the BIODIFF diffractometer at the Forschungs-Neutronen­quelle Heinz Maier-Leibnitz (FRM II), Germany and the AR-NW12A beamline at the Photon Factory (PF), Japan, respectively (Yokoyama *et al.*, 2017 ▸). The neutron structure was jointly refined with the X-ray data at 2.2 Å resolution, resulting in an *R*_cryst_ of 18.4% and an *R*_free_ of 22.2% (Table 2 ▸). The asymmetric unit contains one molecule of Hsp72 and ADP, 86 heavy water molecules, one magnesium ion and one sodium ion [Fig. 2 ▸(*a*)]. Our joint XN structure superposed on the previous structure at cryogenic temperature (PDB entry 3atu; Arakawa *et al.*, 2011 ▸) with an r.m.s.d. of 0.28 Å, indicating that the structures at room temperature and cryogenic temperature were comparable. The ADP molecule was clearly observed in the neutron scattering length density map and the electron-density map, although the neutron scattering length density was partially cancelled by neighbouring H atoms [Fig. 2 ▸(*b*)]. While the neutron scattering length density map omitting H and D atoms clearly showed the presence of the H and D atoms of the adenosine moiety, no difference peaks were observed at the phosphate groups, indicating that the phosphate group of ADP was fully deprotonated. The NBDs of Hsp72 isoforms in complex with ADP are often crystallized with an inorganic phosphate ion derived from the expression host (Wisniewska *et al.*, 2010 ▸). However, inorganic phosphate was not observed in our joint XN structure. The inorganic phosphate ions may have been released during crystallization, as a ten-week period was required in order to obtain sufficiently large crystals for the neutron diffraction experiment (Yokoyama *et al.*, 2017 ▸). We found three water clusters (clusters 1–3) in the Hsp72–ADP complex [Fig. 2 ▸(*a*)]. Water cluster 1 was associated with the binding of the phosphate group of ADP. Water cluster 2 was located between sub­domains IA and IB, distant from the ADP-binding site. Water cluster 3 was located at the interface of subdomains IA and IIA near cluster 1, but water clusters 1 and 3 were not connected by hydrogen bonds.

The H/D-exchange ratio could be determined because H/D-exchanged crystals were used. In particular, the H/D-exchange ratios of amide protons are generally useful to assess protein dynamics, rigidity and water accessibility. While the H/D-unexchanged residues (H > 80%) were primarily observed in subdomains IA and IIA, H/D-exchanged residues (D > 80%) were observed throughout the whole protein [Fig. 2 ▸(*c*), Table 3 ▸]. The H/D-unexchanged residues were located in the hydrophobic core in subdomains IA and IIA. The average *B* factors of subdomains IA and IIA were lower than those of subdomains IB and IIB, suggesting that the formation of a hydrophobic core by the unexchanged residues is likely to contribute to the static stability of the subdomains.

### 3.2. ADP binding

**Figure 3:**
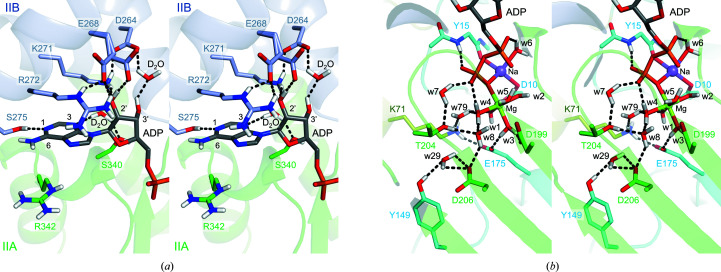
Atomic interactions between Hsp72 and ADP in a wall-eyed stereo representation for (*a*) the adenyl group and ribose moiety and (*b*) the phosphate moiety (PDB entry 7f4x). The C atoms of ADP and subunits IA, IIA and IIB are coloured dark grey, cyan, green and light blue, respectively. H (D), N, O, Na, Mg and P atoms are coloured while, blue, red, purple, yellow green and orange, respectively. The black dashed lines indicate hydrogen bonds.

ADP binds to the NBD with high affinity and the dissociation rate is known to be slow (Höhfeld & Jentsch, 1997 ▸; Arakawa *et al.*, 2011 ▸). The following detailed description of the interactions between Hsp72 and ADP may provide a better understanding of the ADP-binding event. The adenosine moiety is located at the interface of subdomains IIA and IIB. 1-N and 3-N accept hydrogen bonds from Ser275 and a water molecule, respectively, and the water molecule also donates a hydrogen bond to Ser340, bridging the hydrogen-bond network between the adenyl group and Ser340 [Fig. 3 ▸(*a*), Supplementary Fig. S1(*a*)]. 6-NH_2_ is located at the surface, and no specific interactions are observed between 6-NH_2_ and Hsp72. Although the adenyl ring is located in the middle of the guanidyl groups of Arg272 and Arg342, these groups are too far apart to form π⋯π stacking. The ribose is stabilized by hydrogen bonds from 2′-OH and 3′-OH. Glu268 adopts an alternate conformation. One conformation is involved in a salt bridge with the side chain of Arg272 and the other conformation is involved in a hydrogen bond to 2′-OH. 2′-OH also accepts a hydrogen bond from Lys271, which forms a salt bridge with Asp264. 3′-OH donates a hydrogen bond to a water molecule that accepts a hydrogen bond from Asp264. In summary, the adenyl and ribose groups are involved in three and five hydrogen bonds, respectively.

The diphosphate moiety is bound at the interface of subdomains IA, IIA and IIB in a highly hydrated state by water cluster 1 [Fig. 2 ▸(*a*)]. As previously reported, one magnesium ion and one sodium ion are observed. The magnesium ion is octahedrally coordinated by the O atoms of w1–w5 and the β-phosphate group [Fig. 3 ▸(*b*)]. The sodium ion is tetrahedrally coordinated by the O atoms of w6, the side chain of Asp10, the main chain of Tyr15 and the β-phosphate group. Because peaks for the D atoms of the waters could clearly be observed in the neutron scattering length density map, all water molecules coordinate in the form of D_2_O and not DO^−^ [Supplementary Fig. S1(*b*)]. There are three aspartate residues (Asp10, Asp199 and Asp206) and one glutamate residue (Glu175) in the pocket, and they are in deprotonated states. Asp199 accepts a hydrogen bond from w1. w1 also donates a hydrogen bond to Glu175, which forms a salt bridge with Lys71. These residues are considered to be catalytic residues for the ATPase activity. Thr204 accepts a hydrogen bond from w7 and donates a hydrogen bond to w7, participating in the large hydrogen-bond network composed of w4, w7, w8, w79, Lys71 and the β-phosphate group. This hydrogen-bond network expands to Tyr149 via w29 and Asp206. The binding of the diphosphate group to Hsp72 involves more than 20 hydrogen bonds and salt bridges.

### 3.3. Water clusters

Water cluster 1 is composed of four water molecules (w1, w4, w8 and w79) and is located at the binding site of the diphosphate group of ADP [Fig. 3 ▸(*b*)]. Water molecule w4 donates a hydrogen bond to the phosphate group of ADP, and w8 and w79 accept hydrogen bonds from Thr204 and Lys71, respectively. Water molecule w3 donates a hydrogen bond to Glu175. Lys71, Glu175 and Thr204 are catalytically important residues. Water molecules w1 and w4 are coordinated to Mg^2+^. This magnesium ion is also coordinated to w2, w3, w5 and the phosphate O atom of ADP.

**Figure 4:**
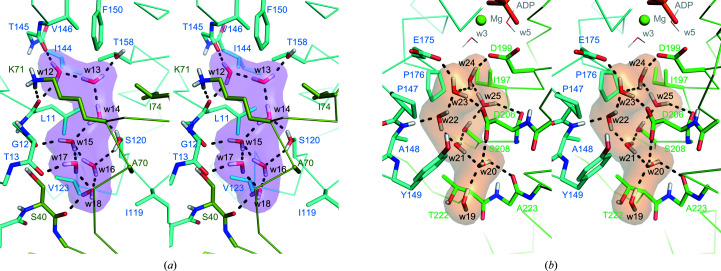
Wall-eyed illustration of the hydrogen-bond networks of (*a*) water cluster 2 (w12–w18) and (*b*) water cluster 3 (w19–w25) (PDB entry 7f4x). The colour scheme is the same as in Figs. 2 ▸ and 3 ▸.

Water cluster 2 is composed of seven well ordered water molecules (w12–w18) with an average *B* factor of 21.4 Å^2^ [Fig. 4 ▸(*a*), Supplementary Fig. S2(*a*)]. Water cluster 2 is mostly surrounded by hydrophobic residues, *i.e.* Leu11, Ala70, Ile74, Ile119, Val123, Ile144, Val146, Phe150 and the hydrophobic moiety of Lys71, and thus the role of the water cluster appears to be to cancel the polarity inside the cavity through hydrogen bonding. While w15, w17 and w18 are hydrogen-bonded in a tetrahedral coordination (two hydrogen bonds as donors and two hydrogen bonds as acceptors), w12, w13, w14 and w16 are hydrogen-bonded in a triangular coordination (two hydrogen bonds as donors and one hydrogen bond as an acceptor). The polarities of the carbonyl O atoms of Leu11, Gly12, Thr13, Ser40 and Thr145 are neutralized by hydrogen bonds donated from w12, w15, w17, w18 and w12, respectively. In addition, w13 donates a hydrogen bond to Thr158. The neutron scattering length density map indicated that the side chain of Ser120 adopts an alternate conformation, donating hydrogen bonds to w14 and w16 [Supplementary Fig. S2(*a*)]. The average hydrogen-bond length between the water molecules in the cluster is 1.93 Å, and the average hydrogen-bond length between the water molecules and the amino-acid residues is 2.03 Å (Supplementary Table S1).

Water cluster 3 is composed of seven water molecules (w19–w25) and they are statically stable with an average *B* factor of 27.0 Å^2^ [Fig. 4 ▸(*b*)]. This water cluster is in contact with the polar residues Glu175, Asp199, Asp206 and Ser208 and the hydrophobic residues Pro147, Ala148, Pro176 and Ile197. Although the three acidic residues are all deprotonated, boomerang-shaped neutron maps are observed for the water molecules, indicating that they are bound in the form of D_2_O and not as hydronium ions [Supplementary Fig. S2(*b*)]. Only w23 is hydrogen-bonded in a tetrahedral coordination, while the other water molecules are hydrogen-bonded in a triangular coordination. The lack of tetrahedral coordination may contribute to the relative instability in comparison to water cluster 2. The D⋯O distance between w24 and w23 is 2.2 Å and is slightly longer than those between the other water molecules (1.9 Å). Glu175 and Asp206 accept hydrogen bonds from w23, and Asp199 accepts a hydrogen bond from w24. This water cluster is stabilized by more than 15 hydrogen bonds and expands to w19, which is located at the entrance to the pocket. Glu175 is considered to be a catalytic residue that supplies a nucleophilic water by deprotonation. The water cluster is likely to stabilize the structure and to supply a nucleophilic water, but not to cancel the extra negative charge of the aspartate and glutamate residues.

It is frequently observed that solvent positions and protein conformations differ between cryogenic and room-temperature structures (Kwon *et al.*, 2018 ▸). Our joint XN structure of Hsp72–ADP was compared with the cryogenic X-ray structures of the Hsp72–AMPPnP (PDB entry 2e8a; Shida *et al.*, 2010 ▸) and Hsp72–ADP (PDB entry 3atu; Arakawa *et al.*, 2011 ▸) complexes. The neutron Hsp72–ADP structure superimposed on the cryo Hsp72–AMPPnP and Hsp72–ADP structures with r.m.s.d.s of 0.39 and 0.28 Å, respectively, indicating that the overall structures are almost identical [Supplementary Table S2, Supplementary Figs. S3(*a*) and S3(*b*)]. In addition, water clusters 2 and 3 were conserved in the cryogenic Hsp72–AMPPnP and Hsp72–ADP structures. In the ADP complex, an inorganic phosphate ion was bound to the ATP-binding pocket and was hydrogen-bonded to Thr204 and Tyr149 via a water molecule, but the presence of a phosphate ion had no effect on the positions of the water molecules in water cluster 3 [Supplementary Fig S3(*b*)]. Collectively, these comparisons indicate that the nucleotides and temperature had no effect on water clusters 2 and 3.

### 3.4. ATPase activity of the Y149A and T204A mutants

Tyr149 is located at the interface of subdomains IA and IIA, and is involved in the conformational change between the ATP-bound and ADP-bound states. Tyr149 participates in the hydrogen-bond network of water cluster 1 and also comes into contact with water cluster 3. The Y145A mutation in DnaK (corresponding to Tyr149 in Hsp72) increased the ATP hydrolysis rate (Kityk *et al.*, 2015 ▸). In addition to our neutron crystallographic analysis, this background prompted us to investigate the role of Tyr149 by determining the ATPase kinetic parameters and X-ray crystal structure of Y149A-mutated Hsp72. We also determined the kinetic parameters of T204A-mutated Hsp72 (T204A-Hsp72). The T204A mutation has been shown to be an ATPase-deficient mutation in DnaK and BiP, and therefore we considered that experiments using T204A-Hsp72 would be suitable as negative-control experiments (Yang *et al.*, 2015 ▸; Qi *et al.*, 2013 ▸).

**Figure 5:**
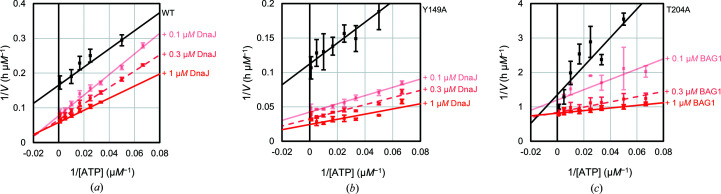
Lineweaver–Burk plots for the ATPase activities of (*a*) WT-Hsp72 in the presence of DnaJB1, (*b*) Y149A-Hsp72 in the presence of DnaJB1 and (*c*) T204A-Hsp72 in the presence of BAG1. Standard error bars and regression lines are drawn based on the mean of five independent experiments.

We adopted the Lineweaver–Burk plot to determine the kinetic parameters (*K*_m_ and *k*_cat_) for full-length Hsp72 using the malachite green assay, in which orthophosphate ions can be directly detected. The Y149A mutation caused an increase in *k*_cat_ and had no effect on *K*_m_ (Fig. 5 ▸, Table 4 ▸). The T204A mutation decreased *k*_cat_ and increased *K*_m_. In addition to determination of the basal activity, we also determined the kinetic parameters in the presence of the co-chaperones Hsp40 (DnaJB1) or BAG1. The *k*_cat_ value of WT-Hsp72 increased in a DnaJB1-dose-dependent manner. The *k*_cat_ and *K*_m_ values decreased in a BAG1-dose-dependent manner and the ATPase activity (*k*_cat_/*K*_m_) was not significantly changed. The *k*_cat_ value of Y149A-Hsp72 was also increased by the addition of DnaJB1, but the stimulation of the ATPase activity was much stronger than in WT-Hsp72. On the other hand, the ATPase activity of T204A-Hsp72 was not stimulated by DnaJB1. The presence of BAG1 drastically decreased the *K*_m_ value and slightly increased the *k*_cat_ value, recovering the ATPase activity to a level comparable to that of WT-Hsp72. In summary, Y149A-Hsp72 and T204A-Hsp72 are more sensitive to DnaJB1 and BAG1, respectively.

### 3.5. X-ray crystal structures of Y149A-Hsp72 in complex with ADP and AMPPnP

X-ray crystal structures of the NBD of Y149A-Hsp72 in complex with ADP and AMPPnP at cryogenic temperature were solved at 1.8 and 1.7 Å resolution, respectively (Table 1 ▸). The Y149A-Hsp72 crystals were isomorphous to WT-Hsp72 crystals in space group *P*2_1_2_1_2_1_. The overall structures of the cryogenic Y149A-Hsp72–ADP/AMPPnP complexes were identical to the room-temperature WT-Hsp72–ADP and cryogenic WT-Hsp72–ADP/AMPPnP structures, with r.m.s.d. values of 0.21–0.38 Å, indicating that the mutation had no effect on the overall structure (Supplementary Table S2).

**Figure 6:**
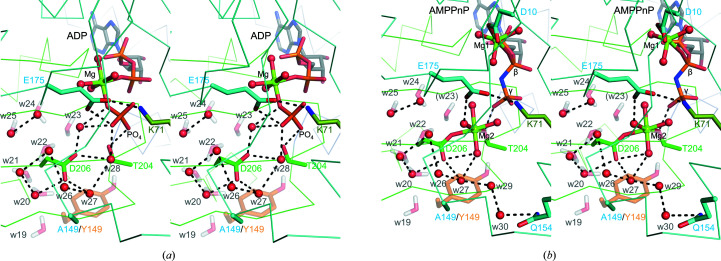
X-ray crystal structures of (*a*) Y149A-Hsp72 in complex with ADP (PDB entry 7f4z) and (*b*) Y149A-Hsp72 in complex with AMPPnP (PDB entry 7f50). The colour scheme is the same as in Figs. 2 ▸ and 3 ▸. The side chain of Tyr149 and water cluster 3 of the superposed joint XN structure (PDB entry 7f4x) are shown as a semi-transparent stick model.

As in the WT-Hsp72–ADP structure, an inorganic phosphate ion was observed in the Y149A-Hsp72–ADP structure [Fig. 6 ▸(*a*), Supplementary Fig S4(*a*)]. This ion was hydrogen-bonded to Glu175 and Thr204 and was coordinated to the magnesium ion. While the water molecules in cluster 3 are conserved in Y149A-Hsp72–ADP, except for w19, which is located at the molecular surface, the network of hydrogen bonds is reorganized due to the movement of w23. w23 is hydrogen-bonded to the phosphate ion and Asp206. The Y149A mutation introduced three water molecules (w26–w28), which form an alternative hydrogen-bond network composed of w20–w22, w26–w28, Asp206 and the phosphate ion.

Two magnesium ions (Mg1 and Mg2) are found in the Y149A-Hsp72–AMPPnP structure. Mg1 is hexahedrally coordinated by the β-phosphate of AMPPnP, Asp10 and two water molecules [Fig. 6 ▸(*b*)]. The position of Mg1 in Y149A-Hsp72–AMPPnP is slightly different from that in WT-Hsp72–AMPPnP. Mg1 in WT-Hsp72–AMPPnP is tetrahedrally coordinated by the β-phosphate of AMPPnP, Asp10, the carbonyl group of Tyr15 and a water molecule [Supplementary Fig. S3(*a*)]. The additional magnesium ion (Mg2) is only found in the Y149A-Hsp72–AMPPnP structure. Mg2 is octahedrally coordinated by Thr204, Asp206 and four water molecules. While w20–w22, w24 and w25 are conserved in the Y149A-AMPPnP structure, w23 was ejected by Mg2, disrupting water cluster 3. The Y149A mutation induced the formation of a hydrogen-bond network composed of w20–w22, w26, w27, w29, w30, Gln154 and Asp206. These hydrogen bonds would stabilize the binding of Mg2. Water molecules w31 and w32, which are coordinated to the magnesium ion, are hydrogen-bonded to Glu175 and the γ-phosphate of AMPPnP. The additional magnesium ion was not observed in the cryogenic WT-Hsp72–AMPPnP structure. The proximity of the magnesium ion to Glu175 and the γ-phosphate group would be responsible for the increased ATPase activity of Y149A-Hsp72.

## 4. Discussion

The ATPase activity of Hsp70s is basally low and is highly controlled by co-chaperones, such as Hsp40s and NEFs, and the SBD. As the molecular mechanism of the intrinsically low ATPase activity of Hsp70s remains unclear, it is often hard to accurately predict the degree of enzymatic activity from their structures and sequences. Because there are a variety of microenvironmental factors that modulate the enzymatic activity, the full characterization of a catalytic reaction requires multifaceted biochemical and biophysical studies. In the present study, we determined the neutron crystal structure of the NBD fragment of Hsp72 in complex with ADP. The observation and location of hydrogens and deuteriums enabled a description of a more ‘complete’ structure and provided unique insights into the protein properties related to the ATPase activity.

Hydrogen/deuterium-exchange ratio analysis revealed that subdomains IA and IIA were significantly more rigid than subdomains IB and IIB [Fig. 2 ▸(*c*), Table 3 ▸]. These features are very likely to reflect a possible conformational change. The NBD in complex with ADP adopts a closed conformation and the binding of BAG proteins to subdomains IB and IIB of the NBD induces a transformation to an open conformation (Shida *et al.*, 2010 ▸; Sondermann *et al.*, 2001 ▸). Crystallographic analysis of the Hsc70–BAG1 complex revealed that the binding of the BAG protein to Hsc70 resulted in movement of subdomain IIB away from subdomain IB. Subdomains IB and IIB may be intrinsically flexible to accept the binding of BAG proteins.

As a result of careful inspection, we found that ADP and the aspartate residues in the ADP-binding pocket (Asp10, Asp199 and Asp206) were fully deprotonated [Fig. 3 ▸(*b*)]. The deprotonation of the aspartate residues appears to be relevant to the charge balance. There are four positive charges on Na^+^, Mg^2+^ and 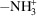 of Lys71 in the ADP-binding pocket. The metal ions are required to increase the electron-withdrawing property of the phosphate group and stabilize the binding of the nucleotide. Lys71 forms a salt bridge with the catalytic residue Glu175 and maintains the deprotonated state of Glu175 that is considered to be necessary to deprotonate a nucleophilic water (O’Brien *et al.*, 1996 ▸; Johnson & McKay, 1999 ▸; Wilbanks *et al.*, 1994 ▸; Flaherty *et al.*, 1994 ▸). While the ATP protonation state in the ATP (or ATP analog) complex structure remains to be elucidated, the deprotonation state of ADP and the acidic residues would stabilize the binding of the metal ions and the protonated state of Lys71.

We characterized water clusters 2 and 3 in the joint XN structure [Figs. 4 ▸(*a*) and 4 ▸(*b*)]. Water cluster 2 was located in a highly hydrophobic pocket that is separated from the nucleotide-binding pocket. In H/D-exchanged crystals, D_2_O molecules in a hydrophobic environment may be blurred due to cancellation effects by the negative scattering signal of H atoms (Fisher *et al.*, 2014 ▸). The water molecules of cluster 2 were clearly observed as a boomerang shape with quite low *B* factors, suggesting that these water molecules were exceptionally stable in the hydrophobic pocket. The formation of water cluster 3 seems to be more relevant to the function of Hsp70 because catalytically important residues such as Glu175, Asp199 and Asp206 are involved in the formation of this cluster (Flaherty *et al.*, 1994 ▸). In addition, Pro147 and Tyr149 hydrophobically enclose the water cluster. This proline residue in DnaK (Pro143) has been suggested to play a key role in allosteric regulation by stabilizing the conformation of the SBD (Vogel *et al.*, 2006 ▸), and Tyr145 in DnaK (corresponding to Tyr149 in Hsp72) also contributes to the allosteric signal by stabilizing the conformation of Pro143 (Kityk *et al.*, 2015 ▸). Our present results clearly showed that the Y149A mutation increased the intrinsic ATPase activity and the sensitivity to Hsp40 (DnaJB1), indicating that Tyr149 is also involved in the catalytic reaction and the stimulation by Hsp40. This tyrosine is also located on the pathway from the binding interface of DnaK and DnaJ (bacterial Hsp40) to the ATPase catalytic centre (Kityk *et al.*, 2018 ▸). X-ray crystallo­graphic analysis revealed that the Y149A mutation disrupted water cluster 3 and caused binding of the additional cation (Mg2) in the AMPPnP complex. Although there is no direct evidence, the physicochemical properties of water cluster 3 may affect the ATPase activity and the stabilization of the allosteric conformation of the SBD.

Crystallographic analysis of Y149A-Hsp72 in complex with AMPPnP elucidated the binding of two magnesium ions (Mg1 and Mg2). Mg1, which is commonly observed in Hsp70 structures, would stabilize the negative charge on the β-phosphate of ATP by its strong electron-withdrawing property. On the other hand, the distal Mg2 would stabilize Glu175 and Thr204. Glu175 is considered to be the general base that deprotonates the nucleophilic water. The proximity of Glu175 and Mg2 would stabilize the deprotonated state of Glu175. Coordination of Mg2 to Thr204 would also stabilize the hydrogen bond between Thr204 and the γ-phosphate. The presence of Mg2 indirectly makes the γ-phosphate more polarized and susceptible to nucleophilic attack by the hydrolyzing water molecule. Taken together, these facts show that Tyr149 hydrophobically positions water cluster 3 and suppresses the incorporation of additional cations, contributing to the intrinsic low ATPase activity. Currently, the crystallization of WT-Hsp72 in complex with AMPPnP for neutron diffraction experiments is under way. The neutron crystal structure determination of the ATP-derivative complex will provide a detailed molecular insight into the enzymatic reaction pathway of Hsp72.

## 5. Conclusions

Our study presents the first neutron crystal structure of Hsp72 in complex with ADP. The joint XN structure provides information on the H/D-exchange ratio that corroborates the previously reported conformational changes. The acidic residues in the nucleotide-binding pocket and the phosphate groups of ADP are observed in their fully deprotonated states. Our neutron crystallographic analysis fully characterizes water cluster 3, which is located near the nucleotide-binding pocket, and suggests that the structural stability of water cluster 3 is associated with the ATPase activity. These results motivated us to investigate the role of Tyr149 using a mutagenic study. The ATPase activity of the Y149A mutant was lower than that of the WT. X-ray crystallographic analysis of Y149A-Hsp72 revealed that the Y149A mutation induces the disruption of the water cluster and the incorporation of an additional Mg^2+^ ion in the catalytic site. These results suggest that Tyr149 contributes to the intrinsically lowered ATPase activity by stabilizing water cluster 3.

## Supplementary Material

PDB reference: nucleotide-binding domain of Hsp72, complex with ADP, joint neutron/X-ray refinement, 7f4x

PDB reference: Y149A mutant, complex with ADP, 7f4z

PDB reference: Y149A mutant, complex with AMPPnP, 7f50

Supplementary Tables and Figures. DOI: 10.1107/S2052252522006297/jt5061sup1.pdf

## Figures and Tables

**Table 1:** X-ray diffraction data-collection and refinement statistics for the cryogenic Hsp72 crystals Values in parentheses are for the outer shell.

	Y149A-Hsp72–ADP	Y149A-Hsp72–AMPPnP
Crystal data		
Beamline	17A, PF	17A, PF
Temperature (K)	100	100
Resolution range (Å)	47.69–1.80 (1.86–1.80)	37.75–1.70 (1.76–1.70)
Space group	*P*2_1_2_1_2_1_	*P*2_1_2_1_2_1_
Unit-cell parameters (Å)	45.9, 63.6, 144.1	45.8, 62.0, 142.8
Observed reflections	215672 (22121)	200742 (18154)
Unique reflections	39659 (3886)	44823 (4381)
Completeness (%)	99.1 (98.5)	98.8 (97.6)
*R*_meas_ (%)	7.6 (70.1)	7.4 (71.3)
*R*_p.i.m._ (%)	3.2 (28.7)	3.4 (33.7)
Multiplicity	5.4 (5.7)	4.5 (4.1)
〈*I*/σ(*I*)〉	16.5 (2.6)	13.9 (2.5)
CC_1/2_	0.998 (0.878)	0.998 (0.771)
Refinement data		
*R*/*R*_free_ (%)	17.8/21.6	18.6/22.7
R.m.s.d., bond lengths (Å)	0.006	0.006
R.m.s.d., angles (°)	0.84	0.81
Average *B* factor (Å^2^)		
Overall	30.6	28.0
Protein	29.8	27.1
Nucleotide	21.7	21.1
Water	38.0	35.9
Ion	25.9	31.9
Ramachandran plot (%)		
Favoured	99.7	99.7
Allowed	0.3	0.3
PDB code	7f4z	7f50

**Table 2:** Statistics for the joint XN refinement of the NBD of Hsp72

	Neutron	X-ray
Source	BIODIFF, FRM II	AR-NW12A, PF
Temperature	Room temperature	Room temperature
Space group	*P*2_1_2_1_2_1_	*P*2_1_2_1_2_1_
*a*, *b*, *c* (Å)	46.7, 64.6, 145.7	46.7, 64.6, 145.6
Resolution range (Å)	39.3–2.20 (2.26–2.20)	44.45–1.60 (1.66–1.60)
No. of unique reflections	20952 (1673)	57639 (5579)
Completeness (%)	90.4 (88.5)	97.5 (95.4)
Multiplicity	2.3 (2.1)	5.7 (5.9)
〈*I*/σ(*I*)〉	8.1 (2.9)	13.5 (2.4)
*R*_meas_	0.117 (0.374)	0.081 (0.733)
Wilson plot *B* factor (Å^2^)	23.3	22.6
Final *R*_cryst_	18.4	16.7
Final *R*_free_	22.2	19.0
No. of non-H atoms	3056	
No. of H/D atoms	2956/841	
No. of waters (D_2_O, DO, O)	74, 9, 3	
R.m.s. deviations		
Bond lengths (Å)	0.007	
Angles (°)	0.99	
Average *B* factors (Å^2^)		
Protein	31.1	
ADP	20.2	
Ion	19.0	
Water	28.4	
Ramachandran plot		
Most favoured (%)	99.5	
Allowed (%)	0.5	
PDB code	7f4x	

**Table 3:** The number of H/D-exchanged and unexchanged residues The numbers in parentheses are percentages of the total number of residues.

Subdomain	No. of residues	H > 80%	D > 80%	Average *B* factor (Å^2^)
Overall	366	64 (17.5%)	178 (48.6%)	31.3
IA	124	38 (30.6%)	50 (40.3%)	28.4
IB	72	2 (2.8%)	37 (51.3%)	32.3
IIA	92	20 (21.7%)	48 (52.2%)	28.8
IIB	78	4 (5.1%)	43 (55.1%)	37.3

**Table 4:** Kinetic parameters for the ATPase activity of WT-Hsp72, Y149A-Hsp72 and T204A-Hsp72 in the presence and absence of co-chaperones

Hsp72	Co-chaperone	*k*_cat_/*K*_m_ (µ*M* ^−1^ h^−1^)	*K*_m_ (µ*M*)	*k*_cat_ (h^−1^)
WT	None	1.4 ± 0.0027	16 ± 1.8	22 ± 0.03
WT	0.1 µ*M* DnaJB1	1.3 ± 0.072	37 ± 5.4	44 ± 3.8
WT	0.3 µ*M* DnaJB1	1.5 ± 0.072	36 ± 4.7	53 ± 4.8
WT	1.0 µ*M* DnaJB1	1.9 ± 0.12	35 ± 5.3	64 ± 6.9
WT	0.1 µ*M* BAG1	1.7 ± 0.28	17 ± 2.7	27 ± 2.2
WT	0.3 µ*M* BAG1	2.2 ± 0.39	13 ± 1.8	25 ± 1.6
WT	1.0 µ*M* BAG1	1.9 ± 0.26	11 ± 0.78	19 ± 1.7
Y149A	None	2.1 ± 0.43	19 ± 3.1	35 ± 3.0
Y149A	0.1 µ*M* DnaJB1	5.8 ± 0.78	14 ± 2.4	79 ± 5.5
Y149A	0.3 µ*M* DnaJB1	6.8 ± 0.99	16 ± 2.7	100 ± 4.8
Y149A	1.0 µ*M* DnaJB1	8.9 ± 0.32	15 ± 2.2	140 ± 16
Y149A	0.1 µ*M* BAG1	2.3 ± 0.50	18 ± 4.2	37 ± 1.7
Y149A	0.3 µ*M* BAG1	1.3 ± 0.12	14 ± 1.4	19 ± 1.2
Y149A	1.0 µ*M* BAG1	1.9 ± 0.39	7.9 ± 1.3	20 ± 6.9
T204A	None	0.082 ± 0.0061	31 ± 7.1	2.5 ± 0.40
T204A	0.1 µM DnaJB1	0.11 ± 0.020	27 ± 7.5	2.7 ± 0.37
T204A	0.3 µ*M* DnaJB1	0.085 ± 0.098	38 ± 8.1	3.1 ± 0.35
T204A	1.0 µ*M* DnaJB1	0.15 ± 0.036	26 ± 4.2	3.7 ± 0.17
T204A	0.1 µ*M* BAG1	0.23 ± 0.041	12 ± 1.6	2.8 ± 0.11
T204A	0.3 µ*M* BAG1	0.46 ± 0.10	9.0 ± 1.0	4.0 ± 0.42
T204A	1.0 µ*M* BAG1	0.88 ± 0.064	4.7 ± 0.31	4.1 ± 0.24
