# Supplementary material for: Neutron crystallographic analysis of the nucleotide-binding domain of Hsp72 in complex with ADP
Source: IUCrJ. 2022 Jul 16;9(Pt 5):562–72. doi: 10.1107/S2052252522006297 (PMC9438496; doi:10.1107/S2052252522006297)
Supplement: Supplementary file 1 [file m-09-00562-sup1.pdf]

IUCrJ

Volume 9 (2022)

Supporting information for article:

Neutron crystallographic analysis of the nucleotide-binding domain of Hsp72 in complex with ADP

Takeshi Yokoyama, Shiho Fujii, Andreas Ostermann, Tobias E. Schrader, Yuko Nabeshima and Mineyuki Mizuguchi

Table S1 Hydrogen bond distances of water cluster 2.

Internal water cluster			Water cluster 2 – Hsp72		
Acceptor	Donor	Distance (Å)	Acceptor	Donor	Distance (Å)
W12−O	W14−D2	1.93	L11−mainchain O	W12−D2	2.06
W13−O	W14−D2	1.90	G12−mainchain O	W15−D1	2.07
W15−O	W14-D1	1.97	T13−mainchain O	W17−D2	1.88
W15−O	W16-D2	1.95	S40−mainchain O	W18−D2	2.02
W17−O	W15−D2	1.89	T145−mainchain O	W12−D1	1.82
W18−O	W16-D1	1.88	T158−O^{γ}	W13−D2	2.03
W18−O	W17-D1	2.02	W14−O	S120−D^{γ}	1.98
			W16−O	S120−D^{γ}	1.98
			W17−O	K71−mainchain D	2.27
Average		1.93	Average		2.03

"Hydrogen bond distances" are between the hydrogen atom and the acceptor atom.

Table S2 RMSD values (Å) of C^{α} distances between the superposed NBD of Hsp72.

RMSD (Å)	WT-ADP neutron @RT (PDB code 7F4X)	WT-AMPPnP @cryo (PDB code 2E8A)	WT-ADP @cryo (PDB code 3ATU)	Y149A-AMPPnP @cryo (PDB code 7F50)	Y149A-ADP @cryo (PDB code 7F4Z)
WT-ADP neutron @RT (PDB code 7F4X)	-	0.39	0.28	0.38	0.38
WT-AMPPnP @cryo (PDB code 2E8A)		-	0.37	0.34	0.35
WT-ADP @cryo (PDB code 3ATU)			-	0.33	0.28
Y149A-AMPPnP @cryo (PDB code 7F50)				-	0.25

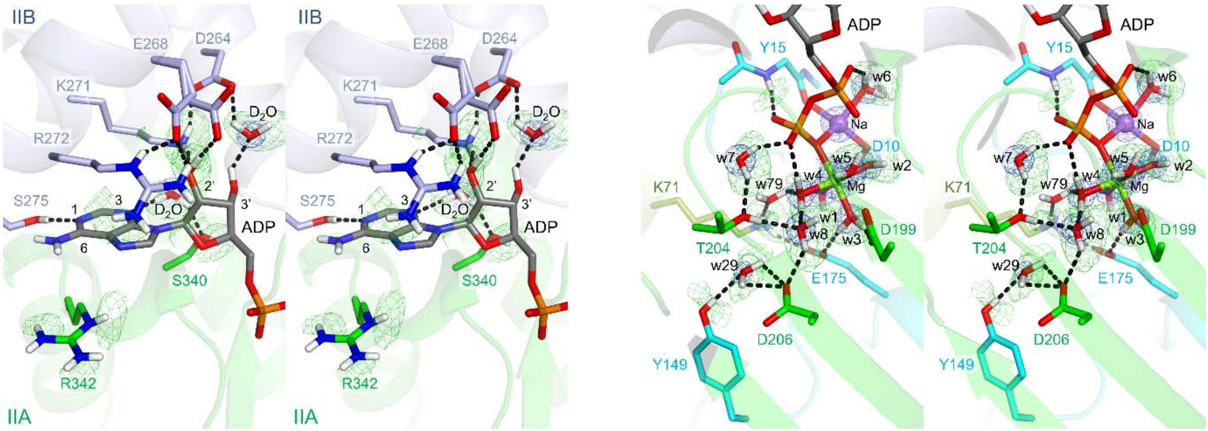

(a) (b)

Figure S1 Neutron and X-ray maps around the adenyl group and ribose moiety (a), and the phosphate moiety (b) (PDB code 7F4X). The $|F_o|$-$|F_c|$ neutron scattering length density map omitting deuterium atoms is contoured at +4σ, and $2|F_o|$-$|F_c|$ electron density map omitting water molecules and sodium ions is contoured at 2σ. The neutron scattering density maps and X-ray electron density maps are indicated as green and blue mesh, respectively.

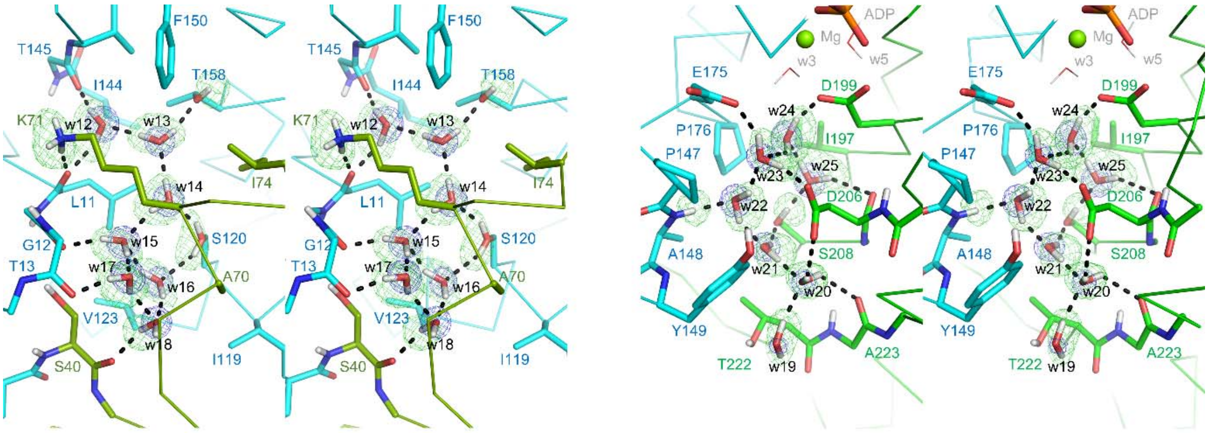

(a) (b)

Figure S2 Neutron and X-ray maps around water cluster 2 (w12-w18) (a) and water cluster 3 (w19-w25) (b) (PDB code 7F4X). The $|F_o|$-$|F_c|$ neutron scattering length density map omitting deuterium atoms is contoured at +4σ, and the $2|F_o|$-$|F_c|$ electron density map omitting water molecules is contoured at 2σ. The neutron scattering density maps and X-ray electron density maps are indicated as green and blue mesh, respectively.

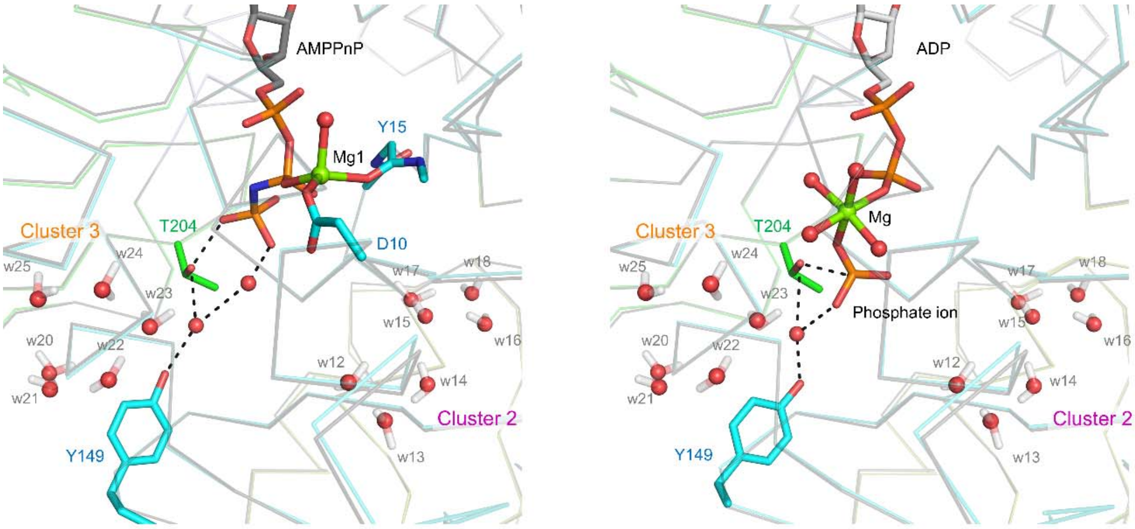

(a) (b)

Figure S3 X-ray crystal structure of Hsp72-AMPPnP (PDB code 2E8A) (a) and Hsp72-ADP (PDB code 3ATU) (b) superimposed on the neutron crystal structure of Hsp72-ADP (PDB code 7F4X). The nucleotide molecules, Y204 and Y149 of the X-ray structure are shown in stick model. The water molecules of the X-ray structure are shown in red sphere model. The water molecules of the overlaid joint XN structure are shown in semi-transparent stick model.

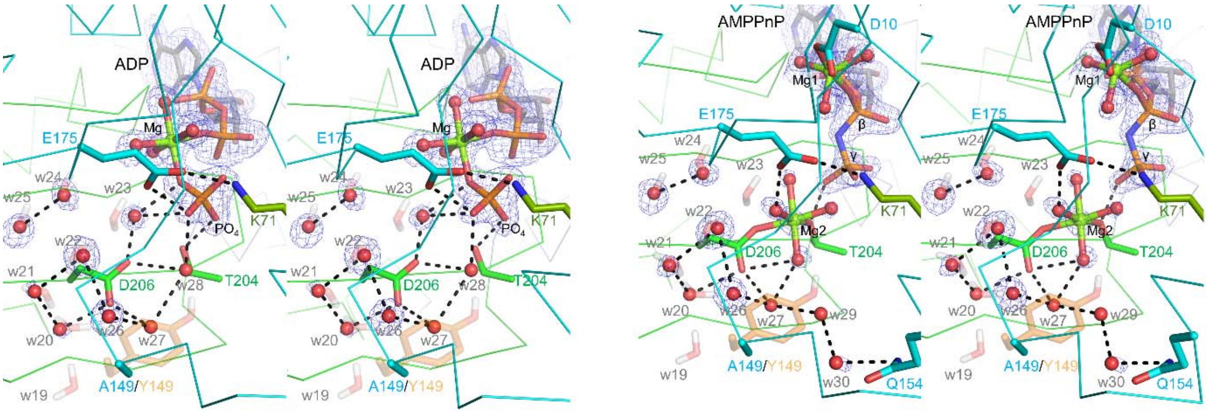

(a) (b)

Figure S4 Omit difference Fourier maps for Y149A-Hsp72 in complex with ADP (PDB code 7F4Z) (a) and Y149A-Hsp72 in complex with AMPPnP (PDB code 7F50) (b). The electron density maps are contoured at 4σ. The side chain of Y149 and water cluster 3 of the superposed joint XN structure (PDB code 7F4X) are shown in semi-transparent stick model. The neutron scattering density maps and X-ray electron density maps are indicated as green and blue mesh, respectively.
